# Alpha Alumina Nanoparticle Conjugation to Cysteine Peptidase A and B: An Efficient Method for Autophagy Induction

**Published:** 2017

**Authors:** Fatemeh Beyzay, Ahmad Zavaran Hosseini, Sara Soudi

**Affiliations:** Department of Immunology, Faculty of Medical Sciences, Tarbiat Modares University, Tehran, Iran

**Keywords:** Autophagy, Cysteine peptidase, Macrophage

## Abstract

**Background:**

Autophagy as a cellular pathway facilitates several immune responses against infection. It also eliminates invading pathogens through transferring content between the cytosol and the lysosomal vesicles and contributes to the cross-presentation of exogenous antigens to T lymphocytes *via* MHC class I pathway. Autophagy induction is one of the main targets for new drugs and future vaccine formulations. Nanoparticles are one of the candidates for autophagy induction. Cysteine Peptidase A (CPA) and Cysteine Peptidase B (CPB) are two members of papain family (Clan CA, family C1) enzyme that have been considered as a virulence factor of Leishmania (L.) major, making them suitable vaccine candidates. In this research, Leishmania major cysteine peptidase A and B (CPA and CPB) conjugation to alpha alumina nanoparticle was the main focus and their entrance efficacy to macrophages was assessed.

**Methods:**

For this purpose, CPA and CPB genes were cloned in expression vectors. Related proteins were extracted from transformed *Escherichia coli (E. coli)* and purified using Ni affinity column. Alpha alumina nanoparticles were conjugated to CPA/CPB proteins using Aldehyde/Hydrazine Reaction. Autophagy induction in macrophages was assessed using acridine orange staining.

**Results:**

CPA/CPB protein loading to nanoparticles was confirmed by Fourier Transform Infrared Spectroscopy. α-alumina conjugated CPA/CPB antigen uptake by macrophages at different concentrations was confirmed using fluorescence microscope and flowcytometry. Highly efficient CPA/CPB protein loading to α-alumina nanoparticles and rapid internalization to macrophages introduced these nanocarriers as a delivery tool. Acridine orange staining demonstrated higher autophagy induction in CPA/CPB protein conjugated with α-alumina nanoparticles.

**Conclusion:**

α-alumina nanoparticles may be a promising adjuvant in the development of therapeutic leishmania vaccines through antigen delivery to intracellular compartments, induction of autophagy and cross presentation to CD_8_ lymphocytes.

## Introduction

Autophagy as a cellular pathway facilitates several immune responses against infection. It also eliminates invading pathogens through transferring content between the cytosol and the lysosomal vesicles. An interaction between autophagy and innate immune signaling pathways modulates inflammatory cytokine productions in response to microbial antigen stimulation ^1^. Autophagy contributes to the cross-presentation of exogenous antigens to T lymphocytes *via* MHC class I pathway. Therefore, due to its anti-microbial benefits, autophagy induction is one of the main targets for new drugs and future vaccine formulations ^2,3^. Nanoparticles are one of the candidates for autophagy induction. A number of studies have reported changes in the basal level of autophagy ^4,5^. Different mechanisms were used to induce autophagy by different nanoparticles ^6^, as reviewed carefully by Panzarini *et al*. The most common mechanism is interaction of nanoparticles with either p62 protein during ubiquitination or aggregated intracellular protein that mimics the invading pathogen and activates LC3 pathway ^6^. Beside the functional properties of nanoparticles, they have gained significant attention in recent years due to the nano-sized structures that are taken up easily by cells ^7^, indicating nanoparticles are also ideal candidates to deliver low-molecular-weight proteins, like antigens. Bioconjugation of nanoparticles with proteins is considered as an efficient method for simultaneous transport to cells and control of the number of protein molecules per Nano-particulate Carrier (NPC) ^7^. This technique is also applied to label the functional groups on biomolecules using either another type of biomolecule or synthetic probes. Cysteine Peptidase A (CPA) and Cysteine Peptidase B (CPB) are two members of papain family (Clan CA, family C1) enzyme that have been considered as a virulence factor of *Leishmania* (*L.*) *major*, making them suitable vaccine candidates. Some studies have also shown that autophagy plays an important role in clearance of the parasite including *L.* species ^8^. Nanoparticles include a variety of materials, like dendrimers, solid lipids, polymers, silicon/carbon materials and magnetic nanoparticles ^1,2^.

Some nanoparticles like polyamidoamine (PAMAM) ^9^, gold ^10^, iron ^11^ and buckminsterfullerene (C60) ^12^ are applied for autophagy induction after being exposed to cells. Furthermore, a study has indicated the key role of aluminium oxide or alpha-alumina (α-alumina/α -Al_2_O_3_) in efficient delivery of antigens to the antigen-presenting cells using autophagosomes, which is then presented to T cells through autophagy ^13^. In the current study, it was aimed to describe the exact steps of conjugation method and characterization of conjugated products. Furthermore, an attempt was made to evaluate autophagy induction by chemo-selective ligation of CPA and CPB with α-alumina nanoparticles in peritoneal macrophages of BALB/c mice using cytotoxicity assay.

## Materials and Methods

The material in this study included GF-1 plasmid DNA extraction kit (Vivantis CO., Malaysia), Clone JET PCR Cloning Kit (Thermo Fisher Scientific, Inc., USA), pET28a, expression vector (Novagen, USA), IPTG (Sinaclon, Iran), kanamycin (Sigma, Germany), Coomassie Brilliant Blue R-250 (Thermo Fisher Scientific, USA), Ni–NTA agarose (Qiagen, USA), Anti-His antibody (Bioligand, USA), IgG-horseradish peroxidase (HPR)-conjugated secondary antibody (Abcam, USA), EDC (G-Biosciences, USA), SANH (Bioworld, USA), FITC (Sigma-Aldrich, USA), 3-MA (Sigma, Germany), and rapamycin (Sigma, Germany).

### Ethical consideration

In this research study, BALB/c mice were used and kept according to the Guide for the Use and Care of Laboratory Animals as approved by the Ethics Committee of Tarbiat Modares University. This study was conducted at Tarbiat Modares University, Tehran, Iran.

### Cloning of CPA and CPB genes in expression vector

*L. major* promastigotes (MHRO: IR: 75: ER) were isolated from infected BALB/c mice as described by Rafati *et al* ^14^ and then used for RNA extraction and cDNA synthesis. The CPA and CPB related coding sequences were amplified by CPA and CPB forward and reverse primers

CPA F: 5/GAC**GGATCC**GCCCCCAGTGGTGTGAT GTCG-3/,
CPA R: 5/GGG**AAGCTT**CTAGGCCGTTGTCGTCG GCAC-3)
CPB F: 5/CG**GGATCC**GATGCGGTGGACTGGCGC GA-3/,
CPB R: 5/GCGC**AAGCTT**CTACACGTACTGGCAA ATG-3/).

In all the above-mentioned primers, BamHI (forward) and Hind III (reverse) were used as restriction sites (represented as bold letters). The following steps were then taken for the amplification program: 98°*C* for 1 *min*, 60°*C* for 2 *min* and finally 72°*C* for 1 *min* for 35 cycles ^14^. The CPA (650 *bp*) and CPB (950 *bp*) blunt-ended PCR products were gel purified using GF-1 plasmid DNA extraction kit (Vivantis CO., Malaysia) and cloned in pJET1.2 plasmid vector using CloneJET PCR Cloning Kit (K1231; Thermo Fisher Scientific, Inc., USA) according to the manufacturer’s protocol. Recombinant pJET1.2 plasmids were verified using common colony-Polymerase Chain Reaction (PCR) and restriction enzyme digestion followed by DNA sequencing. CPB- and CPA-coding DNA was then sub-cloned by insertion between BamHI and HindIII sites in pET28a (expression vector) (Novagen, USA). Constructed recombinant plasmids were subsequently verified by PCR, restriction enzyme digestion and DNA sequencing.

### Recombinant protein expression and purification

Recombinant vectors, pET28a-CPB and -CPA, were introduced into *Escherichia coli (E. coli)* BL21 (DE3) expression hosts. Expression of recombinant CPA and CPB (rCPA and rCPB) was induced by adding 1 *mM* isopropyl-l-thio-fl-D-galactopyranoside (IPTG) (Sinaclon, Iran) and kanamycin (Sigma, Germany) to the culture media at an initial Optical Density (OD) of 0.7 at 600 *nm*. One-four *hr* after the induction, the samples were screened and analyzed by sodium dodecyl sulfate polyacrylamide gel electrophoresis (SDS-PAGE; Bio Rad, Germany) with 12.5% resolving gel, followed by Coomassie Brilliant Blue R-250 (Thermo Fisher Scientific, USA) staining ^15^. Cultures were pelleted, washed with Phosphate Buffer Saline (PBS), and suspended in lysis buffer. Suspensions were sonicated for 3×5 *min* in 0.6 *s* pulses of 80% amplitude. Cell lysates were centrifuged at 18,000 *g* for 20 *min* at 4°*C* to separate soluble (clear lysate) and solid materials (inclusions) ^15^. For purification of the rCPA and rCPB from inclusions, each pellet was dissolved in 8 *M* urea buffer (pH=8) and the debris was removed by centrifugation. Subsequently, 1 *ml* Ni–NTA agarose (50%) (Qiagen, USA) was added to each cell lysate (5 *ml*) and incubated on a rotary shaker at room temperature for 1 *hr*. The mixture was loaded onto a column, and resin was washed twice with four volumes of 8 *M* buffer urea at pH=6.3 and at pH=5.9. Finally, rCPA and rCPB were eluted with elution buffer (pH=4.5). The eluted proteins were analyzed by sodium dodecyl sulfate polyacrylamide gel electrophoresis (SDS-PAGE) and western blot analysis^15^.

### Removal and resolubilization of rCPA and rCPB

To attain a higher concentration of the purified rCPA and rCPB and urea exclusion, on-column resolubilization was applied using a series of wash-buffers with decreasing urea concentration. This procedure was based on purification under hybrid conditions, suggested by Invitrogen (Ni-NTA Purification System; Invitrogen, USA) ^15^.

### Western blot analysis

Proteins (rCPA and rCPB) were separated on 12% gels (Bio-Rad Laboratories, USA) and then transferred to nitrocellulose membranes in a semi-dry transfer cell (Trans-blot SD; Bio-Rad, Germany). The transfer buffer was composed of 25 *mM* Tris-base (pH=8.3), 192 *mM* glycine, and 15% methanol. Following transfer, membranes were blocked for 2 *hr* at room temperature using Tris Buffered Saline (TBS; 10 *mM* Tris-HCL pH=7.6, 150 *mM* NaCl) containing 4% skim milk that was followed by overnight incubation at 4°*C* with Anti-His antibody (1:5000 dilution; Bioligand, USA). Membranes were washed five times with TBST (TBS, 1% Tween-20), incubated for 2 *hr* in anti-mouse IgGHorseradish Peroxidase (HPR)-conjugated secondary antibody (Abcam, USA), and diluted in TBST solution at 1:10 000 dilution. Unbound secondary antibody was removed by washing three times with TBST. Enhanced Chemiluminescence (ECL) was used as substrate.

### α-alumina particles-conjugated with CPA and CPB

α-alumina particles were purchased from US Research Nanomaterials, Inc., USA (Purity: 99+%; Stock number: US3008; 80 *nm*). Firstly, α-alumina particles were functionalized with carboxyl group. For this purpose, suspensions of α-alumina particles were prepared by mixing 1 *g* of particles with 20 *ml* of water followed by sonication for 30 *min* to break agglomerates, while succinic acid was added to the particle suspensions at 0.1 *M*, stirred for 60 *min* at 25°*C*, and then heated for 120 *min* at 100°*C*. Afterwards, the particles were centrifuged for 30 *min* at 1500 *g*. The supernatant was discarded and the particles were freeze-dried. After preparation of functionalized α–alumina particles, three following steps were taken to reach the end product.

### Step 1: Modification of α-alumina particles using benzaldehyde compound

About 4 *mg* 1-ethyl-3-(3-dimethylaminopropyl) carbodiimide hydrochloride (EDC; G-Biosciences, USA) were dissolved in 100 *μl* Dimethyl Formaldehyde (DMF), added to the α-alumina nanoparticle containing carboxyl group and stirred at 25°*C*. The solution was added slowly to the combination of aminobenzaldehyde and stirred for 24 *hr* at 25°*C*. Afterwards, the particles were centrifuged for 30 *min* at 1500 *g*, while the supernatant was discarded and the cell pellets were freeze-dried.

### Step 2: Modification of CPA and CPB proteins using hydrazine reagent

About 2 *mg* succinimidyl 6-hydrazinonicotinate acetone hydrazone (SANH; Bioworld, USA) was dissolved in 100 *μl* DMF. Then, CPA and CPB proteins were added to the above compounds at 0.5 *mg/ml* concentration ^16^.

### Step 3: Modification of CPA and CPB proteins conjugated to α-alumina particles using the aldehyde/hydrazine reaction ^16^

Benzaldehyde-modified α-alumina particles and SANH-modified CPA and CPB proteins were dissolved separately in citrate buffer (100 *mM* sodium citrate, 150 *mM* NaCl, pH=6.0) ^16^. Then, a proper portion of each solution was mixed to another one to obtain a molar ratio with desired properties. The combination was remained for at least 2 *hr* at room temperature.

### Characterization of α-alumina nanoparticle-conjugated with CPA and CPB proteins

To determine the characteristics of nanoparticleconjugated with CPA and CPB proteins, the variations in structure of particles were investigated using Fourier Transform Infrared Spectroscopy (FTIR) and Ultraviolet (UV)-visible spectrophotometer. Transmission Electronic Microscopy (TEM) was used to determine the size and morphology of conjugated and non-conjugated nanoparticles.

### Determination of loading efficiency of α-alumina nanoparticle-conjugated with CPA and CPB proteins

The sample was centrifuged at 3000 *g* for 30 *min* at 4°*C*. The amounts of free CPA or CPB proteins were determined in the clear supernatant using UV-visible spectrophotometry at 280 *nm* and Bradford protein assay. Loading efficacy of α-alumina nanoparticles was determined according to following equation, % Loading= A-B/A×100, where ″A″ is the total amounts of CPA and CPB used for conjugation and ″B″ is the free amounts of CPA and CPB in the supernatant.

### Determination of release potential of α-alumina-conjugated with CPA and CPB proteins

The particular amount of α-alumina-conjugated with CPA and CPB proteins was suspended in separate tubes containing equal volumes of either 0.2 *mol/L* PBS solution (pH=7.4) or citrate sodium buffer (pH=5.4) and incubated by shaking (600 *rpm*) at 37°C. At appropriate time intervals (1, 2, 4, 6, 10, 22, 34, 48, and 72 *hr*), one tube was removed and the sample was centrifuged at 9,000 *g* for 30 *min* at 4°*C*. The amount of protein released into the supernatant was measured according to the following formula, % Release=D/C×100, where ″D″ is the free amounts of CPA and CPB, and ″C″ is the total amounts of CPA and CPB.

### Preparation of FITC-labeled α-alumina-conjugated with CPA and CPB proteins

A solution of at least 2 *mg/ml* of CPA and CPB proteins was prepared in 0.1 *M* sodium carbonate buffer, pH=9. The fluoroisothiocynate (FITC; Sigma-Aldrich, USA) was dissolved in 1 *mg/ml* anhydrous dimethyl sulfoxide (DMSO, Sigma, Germany). For each 1 *ml* of α-alumina-conjugated with CPA and CPB solution, 50 *μl* of FITC solution was added very slowly in 5 *μl* aliquots while stirring the solution gently and continuously. After adding the required amount of FITC, the reaction was continued for 8 *hr* at 4°*C*. Then, 50 *mM* NH_4_Cl was added to the solution and incubated for 2 *hr* at 4°*C*. Finally, xylene cyanol (XC, Sigma, Germany) and glycerol (Sigma, Germany) were added to 0.1% and 5% concentrations, respectively. Free FITCs were separated from the conjugated ones using Amicon filters (Sigma, Germany). The ratio of fluorescein to protein of the product was estimated by measuring the absorbance at 495 and 280 *nm* ^17–19^.

### Peritoneal macrophage preparation and uptake assay

Four days after intraperitoneal (IP) injection of 2 *ml* Brewer thioglycollate medium (4% *w/v*) into BALB/c mice, thioglycollate-elicited macrophages were harvested by IP injection and plated with 10 *ml* cold Roswell Park Memorial Institute (RPMI)-1640 medium (Gibco, USA). Peritoneal exudate cells were cultured at 10-*cm* petri dishes for 6 *hr*. Non-adherent cells were removed, while adherent cells were detached and centrifuged at 400 *g* for 10 *min*. Cells were diluted to 10^4^ *cells/ml* using Dulbecco’s Modified Eagle Medium (DMEM) (Gibco, USA) supplemented with 10% Fetal Bovine Serum (FBS) and cultured at 10^4^ cells/well in 6-well plates ^20^. To assess the uptake capacity of nano-particles-conjugated proteins, FITC-labeled proteins were added to macrophages at 200 *μg/ml* (200 *μg* alpha-alumina containing 16 *μg* CPA and CPB protein), 100 (100 *μg* alpha-alumina containing 8 *μg* CPA and CPB proteins), 10 *μg/ml*, 5 *μg/ml*, as well as 1 *μg/ml* concentrations and incubated for 20 *min* on ice ^21^. Then, unattached particles were washed and macrophages were incubated for 60 *min* at 37°*C* to complete internalization of attached particles. At the end of incubation time, macrophages were analyzed using flow cytometry and a fluorescence microscopy.

### Cytotoxicity assay (MTT Assay)

Cytotoxicity evaluation of α-alumina nanoparticle was performed using 3-(4,5-dimethylthiozol-2-yl)-2,5-diphenyl tetrazolium bromide (MTT) assay which was based on the reduction of the dye MTT to formazan crystals, an insoluble intracellular blue product. Macrophages were cultured at 10^5^ cells/well in 6-well plates and allowed to attach by overnight incubation. Macrophages were treated with series of diluted (0.01, 0.1, 1, 10, 100 and 1000 *μg/ml*) α-alumina nanoparticles for 48 *hr*. At the end of the exposure, 10 *μl* MTT was added to each well to a final concentration of 5 *mg/ml*, and cells were then incubated for 4 *hr* at 37°*C*. The medium was then removed carefully, while 150 *μl* DMSO was added to the cells and the plates were read immediately in a microplate reader (Eppendorf, USA) at 570 *nm*.

### Acridine orange staining: Detection of acidic vesicular organelles (AVO)

Macrophages were cultured at 10^5^ cells/well in 6-well plates and allowed to attach by overnight incubation. Macrophagse were then treated with 100 *μg* α-AL_2_O_3_, 100 *μg* α-AL_2_O_3_-CPA (100 *μg* containing 8 *μg* CPA protein *μg/ml*), 100 *μg* α-AL_2_O_3_-CPA (100 *μg* containing 8 *μg* CPA protein *μg/ml*), 100 *μg* α-AL_2_O_3_-CPB (100 *μg* containing 8 *μg* CPB protein *μg/ml*), 8 *μg* CPA and 8 *μg* CPB, for 48 *hr*. At the end of incubation time, cells were washed twice with PBS and stained with 1 *μg/ml* acridine orange (Invitrogen, USA) in PBS for 15 *min*. Subsequently, the samples were observed under an inverted fluorescence microscope (Zeiss, Germany) ^22^. Also, 3-methyladenine (3-MA; 10 *Mm*, Sigma, Germany) was used as an agent which potently inhibits autophagy-dependent protein degradation and suppresses the formation of autophagosomes, as well as rapamycin ^23^ (50 *nM*; as a control, Sigma, Germany) that is identified as a well-known inhibitor of the PI3K-mTOR pathway and an autophagy inducer ^24^.

### Statistical analysis

One-way analysis of variance (ANOVA) was used to determine the level of statistical significance at p<0.05. All analyses were performed using GraphPad Prism software (GraphPad Software Co., USA) version 5.04.

## Results

### Analysis of CPA and CPB gene cloning

**Figure 1.**
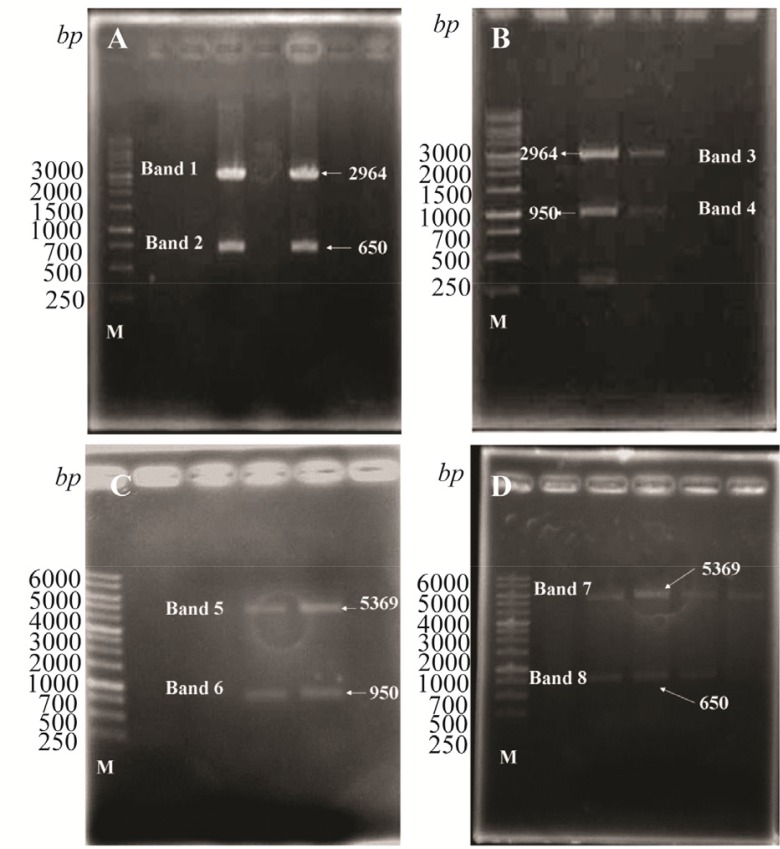
Digestion analysis of pJET1.2-CPA; A) pJET1.2-CPB; B) PET28a-CPA; C) and PET28a-CPB; D) with HindIII and BamH1. Band 1, 2:pJET1.2 (2964 *bp*) and CPA (650 *bp*), Band 3, 4: pJET1.2 (2964 *bp*) and CPB (950 *bp*), Band 5, 6: PET28a (5369 *bp*) and CPB (950 *bp*), and Band 7,8: PET28a (5369 *bp*) and CPA (650 *bp*).

To confirm the CPA and CPB related cDNAs insertion in pJET1.2 vector, DNA was extracted from transformed *E. coli* DH5α and related sequences were amplified using specific primers to both genes. In addition, extracted plasmids were digested using HindIII and BamH1 which created 650 and 950 *bp* fragments for CPB as illustrated in figures 1A and B, respectively. Digestion analysis was also used to determine CPA and CPB subcloned into pET28a expression vector, as shown in figures 1D and C, respectively. Finally, DNA sequencing analysis of rCPA and rCPB plasmids revealed the complete identity with reference sequences, U43706 and U43705.

### Analysis of rCPA and rCPB expression

**Figure 2.**
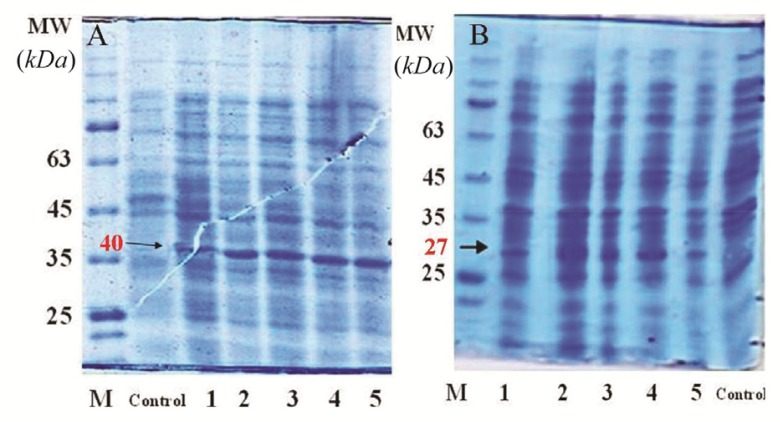
SDS PAGE for crude lyaste of *E. coli* transformed with the PET28a-CPA; A) and PET28a-CPB; B) Control: *E. coli* BL21 strain, lane 1: *E. coli* BL21 strain before induction at time 0, lane 2: *E. coli* BL21 strain one hour after induction, lane 3: *E. coli* BL21 strain two hours after induction, lane 4: *E. coli* BL21 strain three hours after induction, and lane 5: *E. coli* BL21 strain four *hr* after induction.

**Figure 3.**
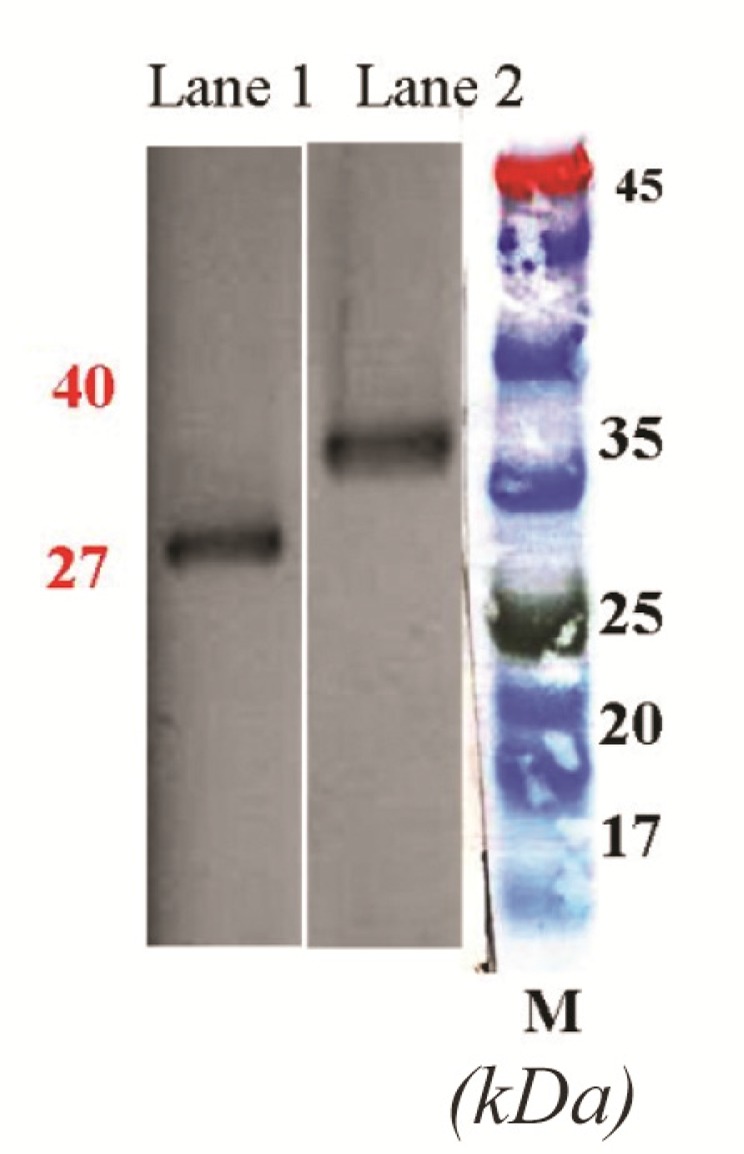
Western blot analysis of L.major CPA and CPB expression in DH5α using HRP-conjugated anti-His tag polyclonal antibody. Lane 1: Purified rCPA, lane 2: Purified rCPB, M: molecular weight marker.

Transformation of pET28a-CPA and -CPB plasmids into *E. coli* BL21 (DE3) strain resulted in the expression of 27 *kDa* and 40 *kDa* proteins after IPTG induction. To validate the expression of CPA and CPB proteins, at different time intervals post IPTG induction, bacterial cell lysates were prepared, while total proteins were extracted and purified using His-tag affinity chromatography. Figure 2 shows the bands of crude protein extract after the transformation of pET28a-CPA and-CPB into *E. coli* BL21 (DE3) at 1 to 6 *hr* post IPTG induction, as determined by SDS*-*PAGE analysis. Figures 2A and B also demonstrate the protein bands at 27 and 40 *kDa* in transformed bacteria as compared to non-transformed *E. coli* that represent the rCPA and rCPB proteins, respectively. After protein purification using Affinity Ni-Charged Resin, western blot analysis was used to confirm recombinant proteins. Anti His-tag antibodies were detected using HRP-conjugated anti-mouse IgG and ECL substrate. Figures 3A and B show the His-tag position on the membrane that reflects the rCPA and rCPB proteins at the same level of 27 and 40 *kDa* protein markers.

### FTIR analysis

**Figure 4.**
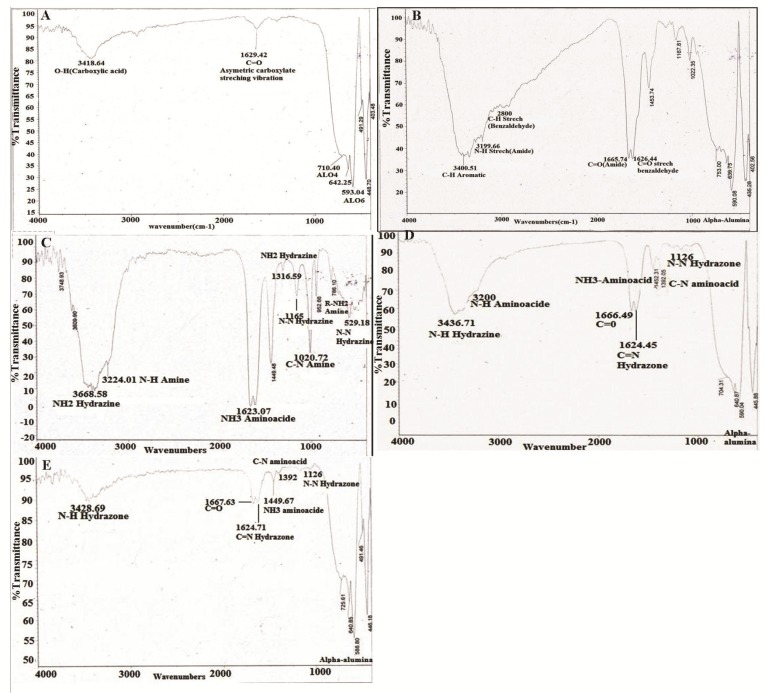
FTIR spectra analysis of; A) α-alumina particles functionalized with carboxyl group, B) Alpha alumina particles modified with benzaldehyde compound, C) CPA proteins modified with hydrazine compound, and (D, E) α-alumina conjugated with CPB and CPA by hydrazone band.

The molecular structure of the α-alumina powder was further confirmed by Fourier Transform-Infrared Spectroscopy (FTIR). An FTIR spectrum was generated by the absorption and transmission of electromagnetic radiation in the frequency range of 400–4000 *cm*^−1^. The α-alumina spectrum is shown in figure 4A. According to FTIR spectra, the bands in the region of 400–1000 *cm*^−1^ were generally associated with stretching vibrations of Al-O. There were some wide and high peaks of Al-O stretching in the range of 500–1000 *cm*^−1^ that were related to the transitional phases of alumina and stable phase of α-alumina. The broad bands at 593 and 710 *cm*^−1^ corresponded to the vibrational frequencies with the internal coordinates of ν-AlO_6_ and ν-AlO_4_, respectively ^25–28^. New alterations were created in FTIR spectrum after different modification in α-alumina structure through new chemical bonding, which suggests a fingerprint for the generated material. As indicated in the FTIR spectra of benzaldehyde (Figure 4B), the bands in the 1626–2850 *cm*^−1^ region were associated with C=O and C-H stretch absorptions for benzaldehyde, while the bands in the 1665–3199 *cm*^−1^ region were related to C=O and N-H stretch absorptions in the amide bond between the carboxyl and amino groups in aminobenzaldehyde ^29^. In figure 4C, the bands in the 1165–3310 *cm*^−1^ region show N-N bond, NH_2_ stretching and NH_2_ wagging in hydrazine bond. Bands in re-gions 529, 786, 1020, 1623, and 3224 *cm*^−1^ indicate C=O, R-NH_2_, C-N, NH_3_, and NH stretch absorptions, respectively, in amino acids of proteins conjugated to α-alumina spectra ^27^ (Figures 4D and E).

### Transmission electron microscopy (TEM)

**Figure 5.**
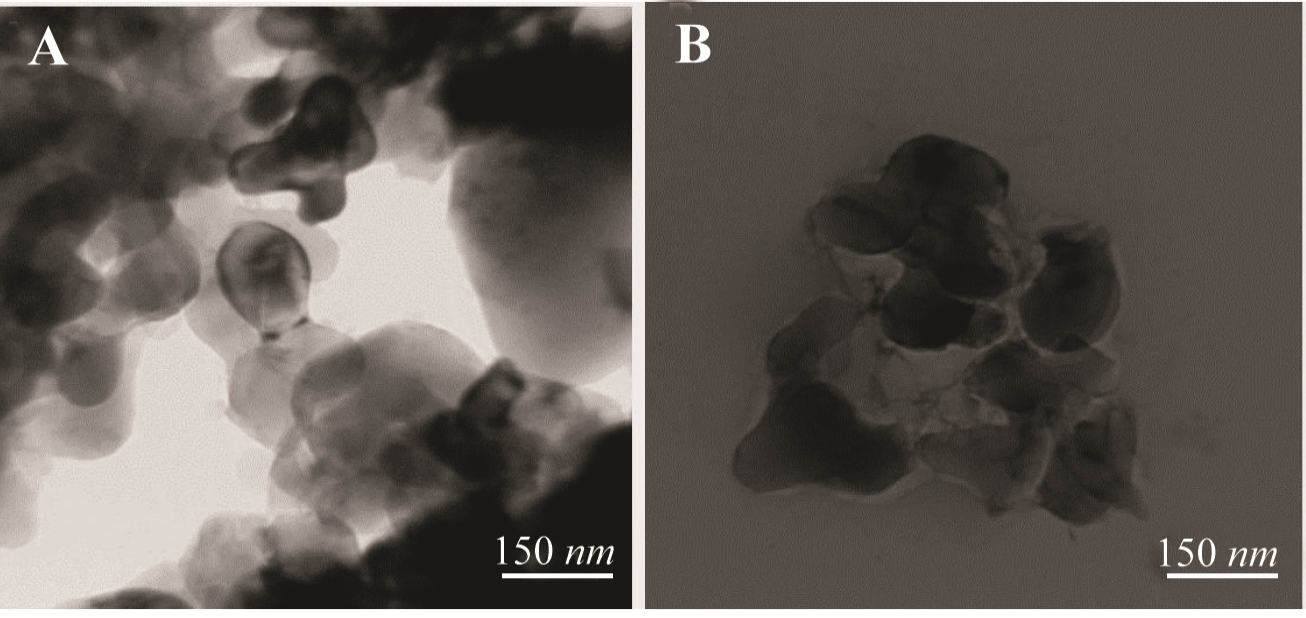
TEM images of α-alumina, A) α-alumina particles modified with benzaldehyde compound (×80,000), and B) α-alumina particles conjugated with CPA, CPB (80,000).

Field Emission-Transmission Electron Microscopy (FE-TEM) was used to identify the morphology of α-alumina nanoparticle. TEM shows agglomerated particles of 80–150 *mm* size (Figure 5).

### UV-visible analysis

**Figure 6.**
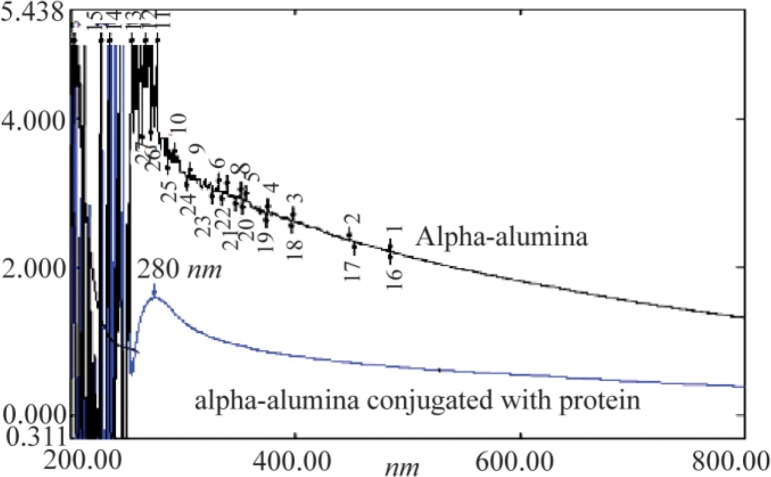
Spectra UV-visible of α-alumina (black) and α-alumina conjugated with CPA, CPB (blue).

Figure 6 shows the UV-visible absorption spectra of AL_2_O_3_ nanoparticles suspended in DMSO. A strong absorption peak at 200–400 *nm* was clearly observed, which confirmed the presence of AL_2_O_3_ nanoparticles. The absorption peak of α-alumina nanoparticles was found at 245 *nm*. CPA and CPB proteins conjugated to α-alumina had an absorption peak at both 242 and 280 *nm*.

### Determination of conjugation efficiency of CPA and CPB proteins conjugated to α-alumina

Different concentrations of α-alumina particles (10–500 *mg/ml*) were used for conjugation to CPA and CPB proteins at 500 *μg/ml*. After conjugation, α-alumina nanoparticles were precipitated and the free amounts of CPA and CPB proteins were determined by Bradford protein assay. According to obtained results, the maximum binding capacity (90%) was observed at 10 *mg/ml* of α-alumina concentration (Table 1).

**Table 1.** The conjugation efficacy of CPA and CPB to α-alumina nanoparticle at different α-alumina concentrations

**Alpha-alumina concentration (*mg/ml*)**	**CPA, CPB concentration (*μg/ml*)**	**Conjugation efficiency:**
500	500	30%
100	500	60%
10	500	90%

### Release studies

**Figure 7.**
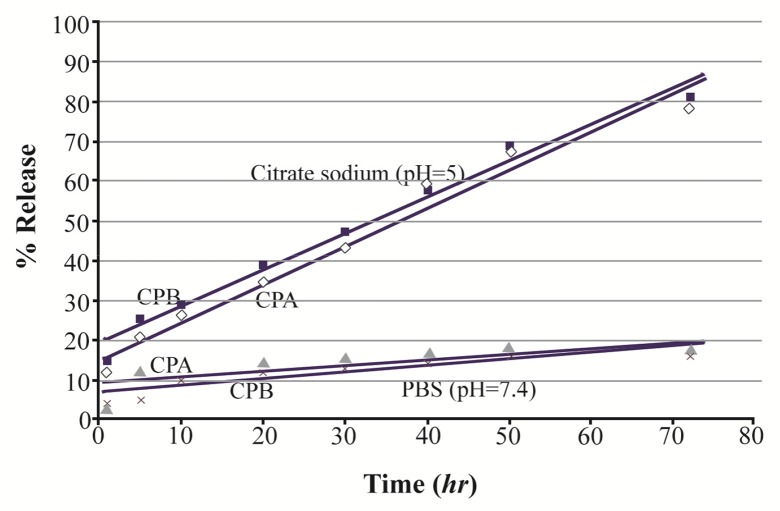
*In vitro* release profile of CPA and CPB from alpha-alumina nanoparticles in PBS (pH=7.4) and citrate sodium buffer (pH=5).

Our observations showed that about 79% and 81% of the loaded CPA and CPB, respectively, were released within 72 *hr* of incubation in citrate sodium buffer. The release profile of CPA- and CPB-loaded nanoparticles exhibits an initial burst release of about 57 and 58%, respectively, in the first 40 *hr,* followed by a slow release of 20% for the subsequent 30 *hr* (Figure 7).

### Internalization potential of α-alumina nanoparticle-conjugated with CPA/CPB

**Figure 8.**
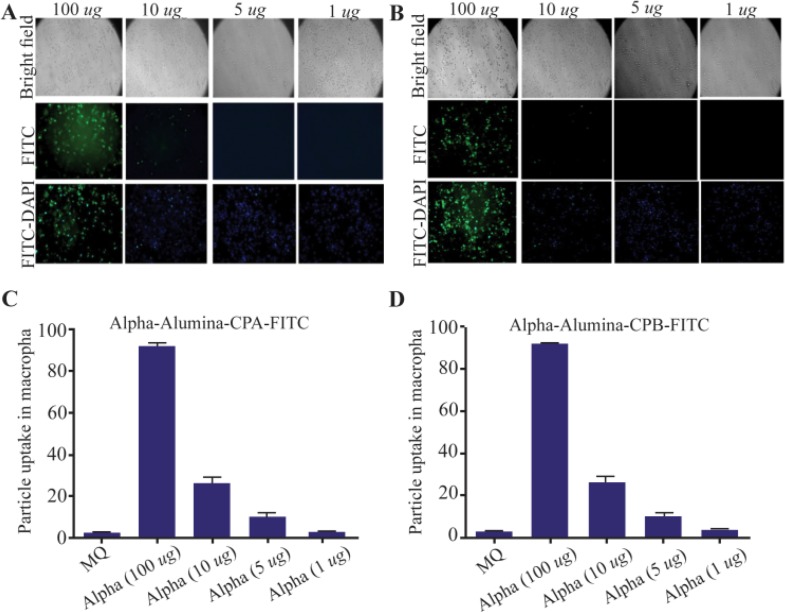
Internalized α-alumina-CPA/CPB-FITC by murine macrophages. A) Fluorescent microscopy of internalized α-alumina -CPA-FITC(A), α-alumina -CPB-FITC(B) by murine macrophages after 30 *min* exposure to 1, 5, 10 and 100 *μg* of different concentrations (×20). C and D show semiquantitative analysis of fluorescent microscopy results by Image J software, 83 and 87% of macrophages received CPA and CPB proteins conjugated to nanoparticles, respectively at 100 *μg/ml* concentration.

**Figure 9.**
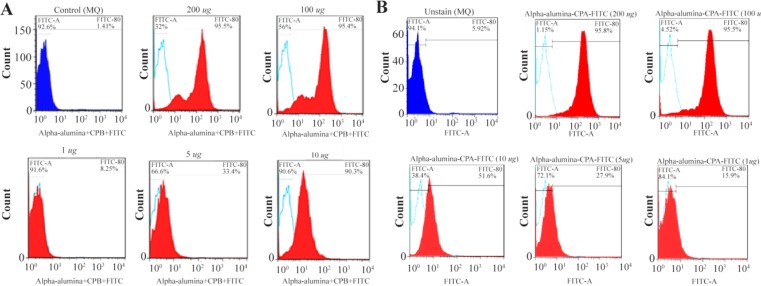
Flowcytometry analysis of internalized α-alumina-CPA/CPB-FITC by murine macrophages. A) Shows that 8.2, 33.3, 90.3, 94.55 and 96.7% of macrophages received α-alumina-CPB-FITC labeled particles in the presence of 1, 5, 10, 100 and 200 *μg/ml* concentrations, respectively. B) Shows that 15, 27, 61, 95 and 98% of macrophages received α-alumina-CPB-FITC labeled particles in the presence of 1, 5, 10, 100 and 200 *μg/ml* concentrations, respectively.

To determine the internalization potential and best concentration of α-alumina nanoparticles in order to internalize all the target cells within 30 *min*, CPA and CPB proteins were labeled with FITC before being conjugated to α-alumina nanoparticles. Peritoneal macrophages were treated with different concentrations of FITC-labeled CPA and CPB conjugated to nanoparticle as explained above. As florescent microscopy results illustrated in figure 8, weak particle internalization was observed at 1, 5 and 10 *μg/ml* concentrations. The fluorescence microscopy images were analyzed using Image J software program. According to semi-quantitative results, 83 and 87% of macrophages received CPA and CPB proteins conjugated to nanoparticles, respectively, at 100 *μg/ml* concentration. Also, for further quantification of the uptake percent, the same treatments were analyzed using flow cytometry method. As demonstrated in figure 9B, 1 and 5 *μg/ml* treated macrophages were represented at 8.2 and 33.3% of FITC-labeled CPA conjugated to nanoparticle, respectively, while the values increased significantly to 90.3%, 94.55% and 96.7% for 10, 100 and 200 *μg/ml*, respectively. There was no significant value between 100 and 200 *μg/ml* concentrations. Similar flow-cytometry results were observed for the internalization potential of FITC-labeled CPB conjugated to α-alumina nanoparticle, as represented in figure 9A.

### Cytotoxicity assessment using MTT assay

**Figure 10.**
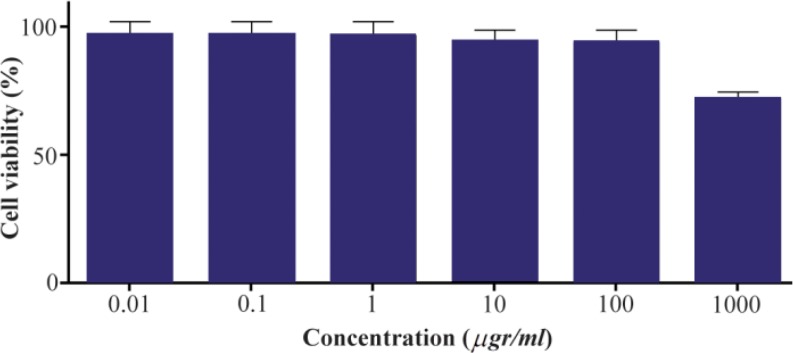
Effect of different concentrations of alpha-alumina nano-particle on macrophage cell viability.

MTT assay was used to examine the cytotoxicity of the α-alumina nanoparticles. Peritoneal macrophages were treated on a medium containing different concentrations (0.01, 0.1, 1, 10, 100, and 1000 *μg/ml*) of the α-alumina nanoparticle. Cell viability was determined at 48 *hr* after treatment. MTT assay showed (Figure 10) more than 95% cell viability at 0.01–100 *μg/ml* concentrations. Cytotoxicity was observed at higher concentrations (≥1000 *μg/ml*) as cell viability reduced to 75% (Figure 10).

### Autophagy induction triggered by α-AL_2_O_3_-CPA and -CPB

**Figure 11.**
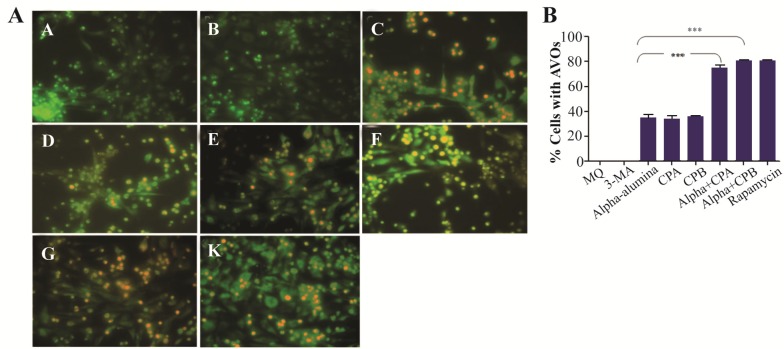
α-alumina-CPA/CPB potently induce autophagy in peritoneal macrophages. After treatment with α-AL_2_O_3_ (D, E), α-AL_2_O_3_-CPA (F), α-AL_2_O_3_-CPB (G), soluble CPA, CPB (K, L), for 48 h, peritoneal macrophages were stained with AO as described in the Material and Methods and detected by fluorescence microscopy. Non-treated macrophage (A), 10 *mM* 3-MA treated macrophage (B) and Rapamycin treated macrophage served as negative and positive controls (C).

In this study, peritoneal macrophages from BALB/c were treated with α-AL_2_O_3_, α-AL_2_O_3_-CPA, α-AL_2_O_3_-CPB, CPA, and CPB and then the presence of autophagy after 48 *hr* was examined using fluorescence microscopy and AO staining. AO as a lysosomotropic dye accumulates in any acidic vacuole in a pH-dependent manner, meaning at acidic environment, AO emits bright red fluorescence ^23,24^. Figure 11 (A–L) depicts AVO or autophagosomes in autophagic cells using AO staining. Figure 11 also shows a significant increase in the intensity of orange color, indicating high amounts of AVOs in α-AL_2_O_3_-CPA (79%) and α-AL_2_O_3_-CPB (70%) as compared to α- AL_2_O_3_ (39%), CPA (35%), and CPB (34%), suggesting that there was a statistically significant increase regarding autophagy in macrophages treated with α-AL_2_O_3_-CPA and -CPB.

## Discussion

NPCs like nanoemulsions are among antigen delivery systems that transport associated antigens into immune cells and provide their stability before reaching the antigen-presenting cells ^30,31^. Thus, new generation vaccines are most likely made of NPCs in conjunction with recombinant antigens and adjuvants ^31^. Different nanoparticles, like carbonhydrate-based formulations, liposomes, poloxamers, emulsions and aluminum-based mineral salts, have been used in delivery of *L. major* antigen to immune system 30,31. In the current study, α-alumina played a key role in autophagy induction through macrophage responses to control *L.* infection. CPB and CPA cysteine proteinases have also been used for conjugation to nanoparticles as a virulence factor of *L. major* in order to produce the new generation vaccines ^32,33^. In the current study, to prepare a reproducible protein source, the whole coding sequence of the CPB and CPA cysteine proteinases of *L. major* was successfully cloned using pET28a (+) vectors. pET28a-CPA and -CPB showed the efficient expression rate in *E. coli* BL21. For purification of His-tagged rCPB and rCPA in the form of inclusion bodies, buffers containing high concentrations of urea (8 *M*) were used. After removing the denaturing agent, the purified proteins in soluble form were achieved. Subsequently, prepared proteins were used for conjugation to α-alumina using a chemoselective ligation method. According to this procedure, α-alumina particles were functionalized initially with carboxyl group and then coupled with aminobenzaldehyde by amide bond formation using EDC. Then, the aldehyde functionalized nanoparticles were conjugated to hydrazine-modified protein. As demonstrated in table 1, different concentrations of α-alumina were applied in conjugation with constant amounts of protein to find better α-alumina concentration for maximum loading of 92 and 83% for CPA and CPB, respectively. To confirm the conjugation process, after each chemical step, a lyophilized sample was analyzed by FTIR method. According to FTIR results, formation of new chemical bonds, especially hydrazone band between protein and α-alumina was confirmed. In addition, UV-visible spectrophotometer was used to analyze proteins conjugated to α-alumina, represented in both black and blue color lines (Figure 6), indicating the absorbance at both 240 *nm* (α-alumina) and 280 *nm* (CPA/CPB). Similar conjugation method has been reported by Li *et al* regarding ovalbumin protein conjugation to α-alumina ^34^. In the current study, peritoneal macrophages were used to evaluate the effects of α-alumina -conjugated with CPA and CPB proteins on autophagy induction. Brewer thioglycollate medium stimulation was used for macrophage preparation because of its effect on monocyte-macrophage migration from blood to peritoneal cavity resulting in more macrophage harvest. Thioglycollate stimulation may also affect macrophage function; however, Brewer thioglycollate formulation has less effect according to the previous studies ^35^. Before nanoparticle application to autophagy induction, the cytotoxic effect was measured and the amount ≤100 *ug/ml* was introduced as non-cytotoxic concentration. This result was in accordance with the study of Dang *et al*, in which they have showed that cells lose their activities at higher concentrations of α-alumina ^36^. Internalization potential of nanoparticles to macrophages at different concentrations was confirmed using a fluorescence microscope and flow cytometry. There was no linear correlation between increased dose of α-alumina and internalized particle. According to our results, 90% of macrophages received particles at concentration of 10 *μg* that was followed by 94.4 and 98% at concentrations of 100 and 200 *μg*, respectively, suggesting that there is no significant difference in this regard among various concentrations of α-alumina. So, the optimum dose for internalization in most cells is 10 *μg*, but the maximum internalization happened at 100 *μg*, according to the Mean Fluorescent Intensity (MFI). No significant difference was seen in MFI between 100 and 200 *μg*, so the concentration of 100 *μg* was used for autophagy assessment. Furthermore, acridine orange staining revealed that α-AL_2_O_3_-CPA and -CPB are potent autophagy inducers. There was no report on the autophagy induction by CPA and CPB in macrophages, but Williams has reported their effects in facilitating effective differentiation in *L. Mexicana* through autophagy ^33^.

Many nanomaterials were reported to change the basal level of autophagy ^5,37^. Chen *et al* have studied the correlation between α-AL_2_O_3_ nanoparticles and mitochondria dysfunction that leads to autophagy ^38^. In another study by Li *et al*, they have showed that α-AL_2_O_3_ nanoparticles at two sizes (60 and 200 *nm*) induce autophagy effectively and exhibit potent anti-tumor capability ^34^. A number of studies have also indicated that alum induces IL-1β *via* the Nlrp3 inflammasome that results in autophagy ^38,39^. In the current study, the mechanism of autophagy induction by nanoparticle was not investigated; however, all the mentioned studies have showed the mechanisms of α-AL_2_O_3_-induced autophagy. Furthermore, due to discovery of diverse effects of new nanoparticles on immune system, further research remains to be done to achieve the desired effects on immune system.

## Conclusion

In this study, α-alumina nanoparticle-conjugated antigen method and characterization of conjugated products were demonstrated. In addition, the products were applied in autophagy measurement. The results confirmed the high efficacy of nanoparticle for antigen delivery and autophagy induction. Activation of autophagy pathway may facilitate antigen cross-presentation that leads to proliferation of antigen-specific CD_8_ T cell that can be a promising effect of nanoparticles in future vaccine formulations. However, further study is necessary to assess the long-term effect.
